# Research Progress in Heterologous Crocin Production

**DOI:** 10.3390/md22010022

**Published:** 2023-12-28

**Authors:** Junjie Zhou, Danqiong Huang, Chenglong Liu, Zhangli Hu, Hui Li, Sulin Lou

**Affiliations:** 1 College of Life Sciences and Oceanography, Shenzhen University, Shenzhen 518060, China; 2200251049@email.szu.edu.cn (J.Z.); dqhuang@szu.edu.cn (D.H.); cl.l@szu.edu.cn (C.L.); huzl@szu.edu.cn (Z.H.); lihui80@szu.edu.cn (H.L.); 2 Shenzhen Engineering Laboratory for Marine Algal Biotechnology, Longhua Innovation Institute for Biotechnology, Shenzhen University, Shenzhen 518060, China

**Keywords:** synthetic biology, crocin, heterologous production, microalgae

## Abstract

Crocin is one of the most valuable components of the Chinese medicinal plant *Crocus sativus* and is widely used in the food, cosmetics, and pharmaceutical industries. Traditional planting of *C. sativus* is unable to fulfill the increasing demand for crocin in the global market, however, such that researchers have turned their attention to the heterologous production of crocin in a variety of hosts. At present, there are reports of successful heterologous production of crocin in *Escherichia coli*, *Saccharomyces cerevisiae*, microalgae, and plants that do not naturally produce crocin. Of these, the microalga *Dunaliella salina*, which produces high levels of *β*-carotene, the substrate for crocin biosynthesis, is worthy of attention. This article describes the biosynthesis of crocin, compares the features of each heterologous host, and clarifies the requirements for efficient production of crocin in microalgae.

## 1. Introduction

**Figure 1:**
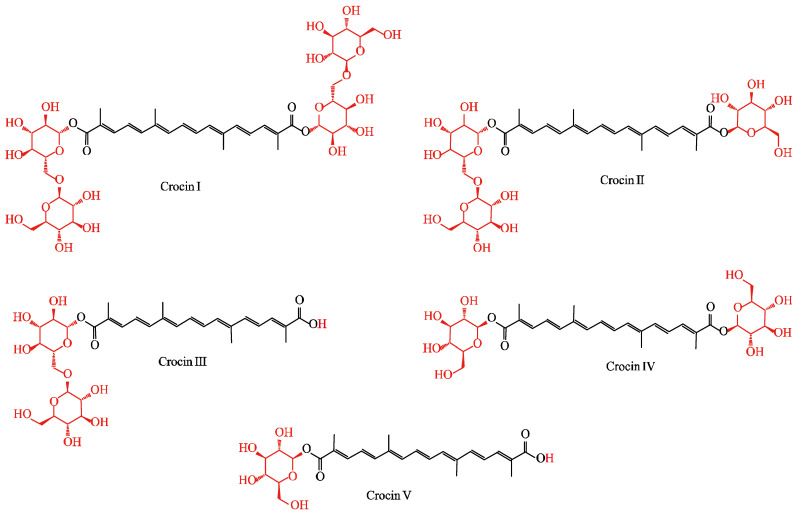
The structures of five crocin variants (from *PubChem*).

Crocin is a well-known aromatic substance produced by plants as a secondary metabolite [1,2,3,4]. It has a polyunsaturated conjugated acid structure with four side-chain methyl groups and seven conjugated double bonds in both *cis*- and *trans*-forms [5]. It is commonly stabilized by esterification with gentiobiose, glucose, or other common sugar moieties [6]. In nature, depending on the number of glucose groups, crocin can be classified into five different derivatives, i.e., crocin I, crocin II, crocin III, crocin IV, and crocin V (Figure 1) [7]. Crocin is a water-soluble carotenoid that is about 13.27 mM at 25 °C and is naturally enriched in the red part of the stigma of *Crocus sativus* L. [8,9,10]. Among the five derivatives, crocin I and crocin II are found in the highest concentrations in the stigma [7,11]. Although different derivatives may have different numbers of glucose groups in various structural arrangements, resulting in diverse chemical properties and catabolic characteristics, their pharmacological effects should be similar since they share the same core structure [7]. Apart from *C. sativus*, crocin is also found at low levels in a few other plants, such as the flowers of *Buddleja davidii* and the fruit of *Gardenia jasminoides* Ellis [5,12,13]. At present, in industry, natural crocin is only extracted from *C. sativus*, which represents an obstacle to large-scale production of crocin.

Crocin is produced naturally from the carotenoid biosynthesis pathway. Carotenoids are an important group of pigments found in plants, algae, bacteria, and fungi that can provide color from yellow to red, depending on their cellular accumulation levels, and can also participate in multiple biological functions, such as light harvesting and photoprotection [14,15]. One of the final products of carotenoid metabolism, crocin has been used in cosmetics, dietary supplements, and medicines; in the latter case, it has been used as an anticancer agent, to reduce the risk of atherosclerosis, and to help prevent Alzheimer’s disease [5,16,17,18,19]. Crocin has become known as “red gold” and is reputed to provide beneficial health effects [20]. Since low yields and a scarcity of natural sources have seriously hindered commercial crocin production, researchers have turned to more efficient heterogeneous production methods using engineered hosts, which show promise for an improved yield of crocin [18,21,22]. Heterologous synthesis of crocin may be a new hope for large-scale industrial production of crocin in the future.

In this study, we review the latest progress on crocin biosynthesis in various host cells, which is a prerequisite for industrial production of crocin in large quantities. In addition, we also discuss the future development of crocin-related products using genetic engineering technology, such as cloning of the target gene, codon optimization, expression vector construction, and different transformation methods. To improve the manufacturing technology, the research of crocin metabolic engineering has attracted increasing attention, and the development of engineered microorganisms and biofortified plants to produce crocin in vivo is the main research direction.

## 2. The Challenge of Crocin Production

The cultivation of *C. sativus*, the main source of crocin, is insufficient to support crocin production. The flowers of *C. sativus* contain three stamens, three pistils, and six petals. In particular, the pistil stigmas account for about 7% of the total mass of the flower and are the main source of bioactive compounds such as crocin, picrocrocin, and safranal [15,23,24]. After separation and drying, the stigmas can be processed into red, filament-like dry products that are widely used as a dye, spice, and traditional medicine [23,25,26,27]. About 75,000 flowers and 200 h of work are required to process one pound of *C. sativus* stigmas [3,28]. In order to preserve the integrity of volatile substances in *C. sativus* as much as possible, farmers have to manually pick the delicate flowers when the stigma is not fully exposed in the bud [29]. Since no mechanical equipment can separate the filamentous pistil stigma, the harvesting and separation stages are the most time-consuming and labor-intensive steps in the entire production cycle [3,30,31]. This greatly limits the yield of crocin and results in a high price in the international market.

In recent years, global climate change has led to flowering problems in *C. sativus* [32] and has been accompanied by desertification of farm land and a reduction in the cultivated area [29]. Moreover, other factors including the lack of high-quality germplasm resources for the improvement and breeding of *C. sativus* [33], damage to bulbs caused by pathogens [34], and unscientific cultivation and management methods [35] have severely hindered the practicability of large-scale production of *C. sativus*; as a result, the price of *C. sativus* has reached an astounding level of USD 40–50 per gram [30]. Many botanists are working to improve the breeding and cultivation techniques of *C. sativus* to increase production. It has been documented that *C. sativus* shows higher heterozygosity owing to its three homologous chromosomes, thus rendering efforts to alter its genome by traditional plant breeding, which follows Mendelian principles, a major challenge [18,36,37]. The breeding of polyploid plants requires the aggregation of each allele to achieve a state of polyploid homozygosity, but achieving this ideal with *C. sativus* would require considerable acreage, a great deal of manpower, and significant financial investment over a long period [37]. Therefore, vegetative reproduction using bulb propagation is traditionally used, with its advantages of a loss of juvenility, rapid development, and a higher rate of growth, and it is conducive to obtaining a large number of propagules with standardized characteristics [38]. However, long-term vegetative reproduction also brings some potential risks, such as reduced genetic diversity of crops in the region and a susceptibility to large-scale infection with diseases like potato late blight and banana bunchy top disease, which have caused huge losses in parts of the agricultural industry [39]. Moreover, the preservation of bulbs is a significant dilemma. Although its incubation at 25 °C is longer than 150 days, the number and size of flowers formed and the yield of saffron per corm depend on the duration and conditions of cold storage, between 0.5 to 2 °C to achieve longer storage time [32]. Traditional farming practices are struggling to sustain the growing consumer demand for crocin until new farming and management technology innovations arrive.

Although the world market is far from being depleted of crocins, the continued adoption of traditional *C. sativus* cultivation is unsustainable [29]. Some unscrupulous merchants use synthetic compounds for dyeing and use other fibrous materials for counterfeiting and doping to deceive consumers, resulting in the market being flooded with various inferior and fake *C. sativus* products, seriously disrupting the normal market and causing great damage to the health and property of consumers [15,40,41]. Due to the high price and chaotic market of *C. sativus* at present, some researchers have proposed introducing several key genes of the crocin biosynthesis pathway into *Escherichia coli*, yeast, or other organisms, alongside optimizing *C. sativus* cultivation techniques and conducting large-scale cultivation in other suitable areas.

## 3. Alternative Sources of Crocins

In China, large-scale cultivation of *C. sativus* has been carried out in Tibet, the most famous plantation base of *C. sativus* in the country, but also in Zhejiang Province and Shanghai City, which are leaders in *C. sativus* industry and technology nationally [42,43]. *C. sativus* contains 150 types of volatile and non-volatile compounds, of which more than 34 are aromatic substances such as volatile terpenes, terpene alcohols, and their esters [3,4]. Crocins, crocetin, picrocrocin, and flavonoids (quercetin and kaempferol) are the main non-volatile components of *C. sativus* [4]. *G. jasminoides* is now commercially exploited as a source of crocin because it represents an inexpensive alternative plant source [44,45,46]. The content of crocin derivatives in its ripe fruits can reach 2.4 mg/g (dry matter) [45,47]. However, some merchants sell cheap *G. jasminoides* material at a high price as if it derived from *C. sativus* since the crocin obtained is similar from both plants, and it is difficult to distinguish the two sources by conventional methods [41,48]. However, this practice still does not meet existing demand because *G. jasminoides* produces far less crocin than *C. sativus*, and the ratio of planting input to output is low [49]. Recently, it was reported that a certain amount of crocin is found in *Freesia hybrida* Klatt, which belongs to the same family, Iridaceae, as *C. sativus* [50]. Of all the plants that are capable of producing crocin, only in *F. hybrida* is there a UDP-glucose transferase capable of constructing a neapolitanosyl group containing three glucose moieties [50]. This study opens the feasibility of freesia yellow flowers as new edible flowers with beneficial functions for human health.

Although crocins are produced naturally in plants, they are very difficult to extract in commercial quantities because of their low concentration, the multiple steps required for isolation and purification, the environmentally unfriendly nature of the process, and the seasonality of flower growth [51]. A huge range of yields are reported for crocins from *C. sativus* in different studies, as shown as Table 1, which may partially depend on production methods; for example, where methods involve a dehydration step by heating, this causes the conversion of picrocrocin to safranal [52]. A comparatively high yield of crocins can be produced by chemical synthesis, but this approach suffers from the formation of many unwanted side products that make purifying the synthetic crocins complicated. Contamination with such side products represents a high risk when using such crocins as medicine or food ingredients [18,21,22]. Different biotechnological approaches, such as tissue culture and genetic engineering, have been applied as alternative bio-sustainable resources for the production of crocins [53,54]. Callus culture, plant culture, hairy root culture, cell suspension culture, genetically modified transgenic plants, and recombinant microbes are well-established methods. For a number of years, many high-value compounds have been produced successfully in microorganisms [55]. In vivo production of natural products in microorganisms has been widely used to address resource shortages for products such as artemisinic acid [56], etoposide aglycone [57], breviscapine [58], and ginsenosides [59,60]. The synthetic biology of crocetin and crocins has attracted a great deal of attention, and genetic engineering, involving the introduction of heterologous pathways from plants into a host strain, has resulted in the bioproduction of crocins [61]. Crocetin and crocin V were successfully produced in engineered *E. coli* and *Saccharomyces cerevisiae*, but these strains showed low productivity [7,61,62,63]. Crocin has also been successfully synthesized in vitro using an enzyme cascade [64]. The authors demonstrated an 80.8% conversion rate of crocetin within 2 h with a yield of 1.48 mg/L/h by optimizing the ratio of enzymes in the system and reducing the accumulation of intermediate byproducts [64]. Clearly, to address the high demand for crocins, there is a need for alternative methods.

## 4. Biosynthesis of Crocin in *C. sativus*

**Figure 2:**
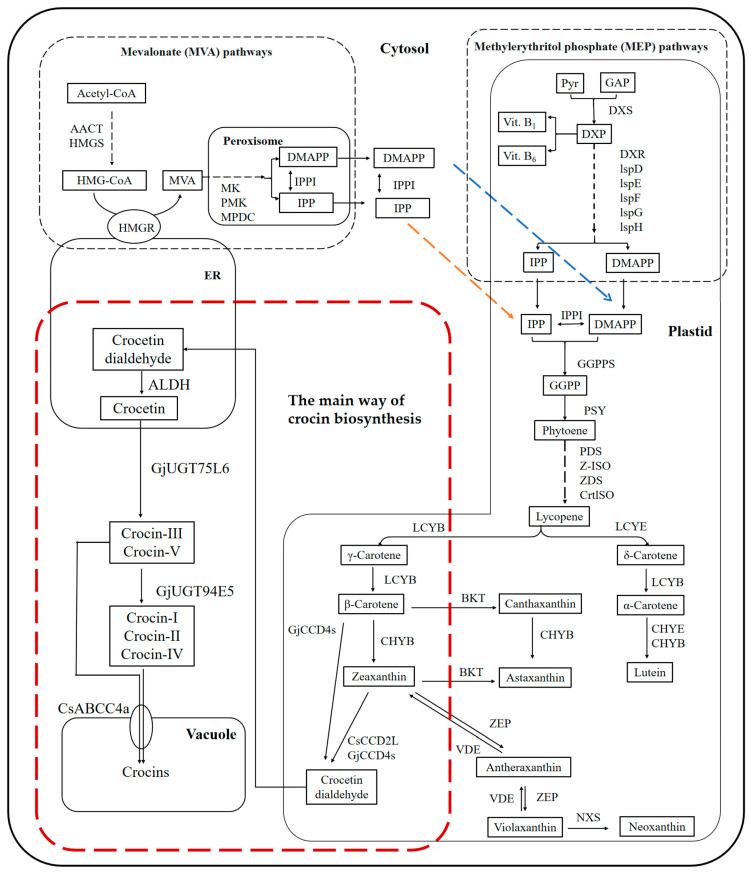
Main crocin biosynthesis pathway for biosynthesis of crocin and other carotenoids. Pyr, pyruvate; GAP, d-glyceraldehyde 3-phosphate; DXP, 1-deoxy-d-xylulose 5-phosphate; DXR, DXP-reductoisomerase; lspD, 4-diphosphocytidyl-2C-methyl-d-erythritol synthase; lspE, 4-diphosphocytidyl-2C-methyl-d-erythritol kinase; lspF, 2C-methyl-d-erythritol 2,4-cyclodiphosphate synthase; lspG, 1-hydroxy-2-methyl-2(E)-butenyl-4-diphosphate synthase; lspH, 1-hydroxy-2-methyl-2(E)-butenyl-4-diphosphate reductase; IPP, isopentenyl diphosphate; DMAPP, dimethylallyl diphosphate; IPPI, IPP isomerase; GGPPS, GGPP synthase; GGPP, geranylgeranyl diphosphate; PSY, phytoene synthase; PDS, phytoene desaturase; Z-ISO, ζ-carotene isomerase; ZDS, ζ-carotene desaturase; CrtlSO, carotenoid isomerase; LCYB, β-cyclase; LCYE, ε-cyclase; CHYE, ε-carotene hydroxylase; CHYB, β-carotene hydroxylase; BKT, β-carotenoid ketolase; ZEP, zeaxanthin epoxidase; VDE, violaxanthin de-epoxidase; NXS, neoxanthin synthase; ALDH, aldehyde dehydrogenase; AACT, acetoacetyl-coa thiolase; HMGS, HMG-CoA synthase; HMG-CoA, 3-hydroxy-3-methylglutaryl-CoA; HMGR, HMG-CoA reductase; MVA, mevalonate; MK, mevalonic acid kinase; PMK, phosphomevalonate kinase; MPDC, mevalonate-5-diphosphate decarboxylase.

The crocin biosynthesis pathway in *C. sativus*, from zeaxanthin to the crocins themselves, lies downstream of the methylerythritol phosphate (MEP) and mevalonate (MVA) pathways, which supply precursors and metabolites to the midstream carotenoid pathway, comprising GGPP to zeaxanthin (Figure 2) [14,70,71,72,73,74,75,76,77,78,79,80,81,82,83,84]. Apart from the astaxanthin synthesis pathway, which is only present in a few specialized microalgae, most algal species in the taxon Chlorophyta share the majority of steps in the carotenoid biosynthesis pathway with higher plants [85]. The production of carotenoids by microorganisms under most conditions uses the isoprenoid precursor isopentenyl pyrophosphate (IPP; C5) along with the allylic isomer dimethylallyl pyrophosphate (DMAPP) [86]. These metabolites are primarily recognized as derivatives of the MVA or 1-deoxyxylulose-5-phosphate (DXP) pathways [86]. Plants and microalgae principally exploit the MVA pathway, while the MEP pathway is primarily used by eubacteria, e.g., *E. coli*, and fungi [77]. The conversion of IPP into DMAPP through isomerization is governed by the enzyme IPP/DMAPP isomerase. Geranylgeranyl diphosphate (GGPP) synthase catalases the reaction that produces the precursor of carotenoid biosynthesis [77]. Altogether, carotenoid biosynthesis mainly consists of three processes, i.e., (1) cyclization, (2) bond migration and hydrogenation followed by the breaking or formation of hydrocarbon chains, and finally (3) methylation or oxygenation; many secondary metabolites are derived from these processes.

**Figure 3:**
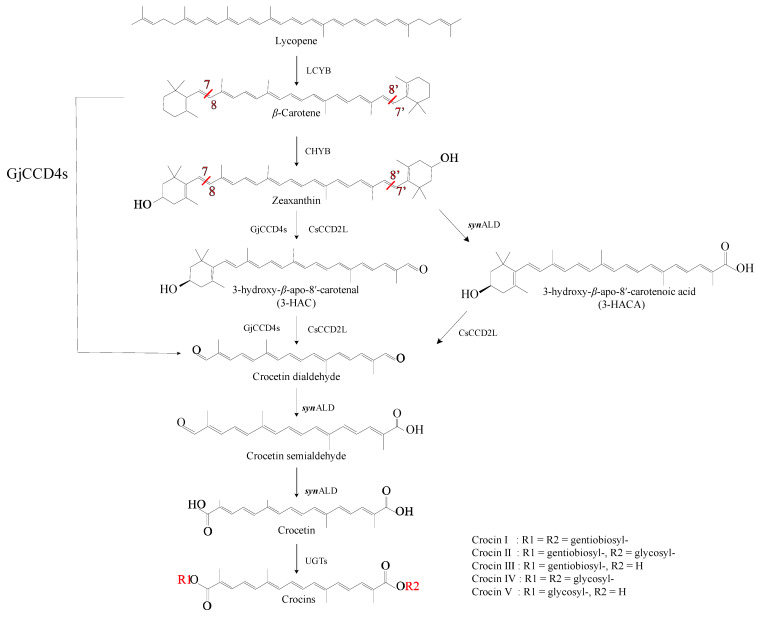
The formation of crocin skeletons.

Lycopene is a natural carotenoid pigment and abundantly found in *Solanum lycopersicum* L. and other vegetables and fruits [87]. It is simultaneously converted into *α*-carotene and *β*-carotene in the carotenoid pathway, with the latter feeding into the crocin biosynthesis pathway. In order to obtain more *β*-carotene to improve the efficiency of crocin biosynthesis, strategies for genetic engineering have focused on lycopene *β*-cyclase (LCYB), which catalyzes lycopene to *β*-carotene, and *β*-carotene hydroxylase (CHYB), which generates zeaxanthin (Figure 3) [88,89]. For example, *Eu-CrtZ*, a gene encoding LCYB from the bacterium *Pantoea ananatis* (reclassified as *Erwinia uredovora*, hence *Eu-CrtZ*), can be expressed in *Yarrowia lipolytica* to obtain 21.98 ± 1.80 mg/L zeaxanthin [89].

Zeaxanthin is a direct precursor in the synthesis of crocins [64]. The downstream pathway mainly consists of three steps. In the first step, the 7, 8 and 7′, 8′ double bonds of zeaxanthin are cleaved by CsCCD2L, which is located in the distal part of the *C. sativus* stigma, to generate crocetin dialdehyde [90]. Zeaxanthin is widespread and abundant in nature and is a cheap raw material for crocins synthesis.

Carotenoid cleavage oxygenases (CCOs) are a class of enzymes that can specifically catalyze the oxidative cleavage of various unsaturated bonds in carotenoids to produce secondary carotenoid metabolites. Depending on the substrate and double-bond specificity, CCOs can be divided into 9-*cis*-epoxycarotenoid cleavage dioxygenases (NCEDs), which generate abscisic acid, and carotenoid cleavage dioxygenases (CCDs), which are involved in the biosynthesis of crocin and other carotenoids [91,92]. Zeaxanthin cleavage dioxygenase (ZCD), which is considered to be the main CCD in *C. sativus*, can cleave zeaxanthin to generate crocetin dialdehyde in the stigma of *C. sativus* [93]. The enzyme of *Cs-ZCD* can cut the double bonds at the 7, 8 and 7′, 8′ positions of zeaxanthin to form crocetin dialdehyde, which initiates biosynthesis pathways for various pigment and aromatic molecules, as well as the crocins [93,94]. However, transcript levels of *Cs-ZCD* do not correlate with changes in the apocarotenoid content of *C. sativus* [95]. Rubio et al. [96] found that *Cs-ZCD* is unable to cleave zeaxanthin and apparently lacks domains that are important for the dioxygenase activity. The same group introduced Cs-ZCD into *E. coli* and maize endosperm also to confirm its inactivity [97]. Therefore, the structure of *Cs-ZCD* is similar to the *N*-terminal moiety of Cs-CCD4a and Cs-CCD4b and thus represents only a partial expression of the CCD4 enzyme rather than a complete CCD; this truncation is the reason for the lack of zeaxanthin-cleaving activity [96]. The endogenous Cs-CCD2L of *C. sativus* can only specifically cleave zeaxanthin to produce crocetin dialdehyde, while Gj-CCD4a from *G. jasminoides* has broader substrate specificity and can directly convert *β*-carotene and zeaxanthin to crocetin dialdehyde [97,98]. It reduced the pathway consumption of *β*-carotene and increased the yield of crocetin dialdehyde. 

The second step in the crocin pathway is the conversion of crocetin dialdehyde to crocetin catalyzed by aldehyde dehydrogenase (ALDH). The enzymes of ALDH are a protein superfamily of NAD(P)-dependent enzymes that oxidize a variety of aliphatic and aromatic aldehydes to nontoxic carboxylic acid molecules [99]. According to reports, among the six highly expressed ALDH enzymes in the stigma of *C. sativus*, only Cs-ALDH3I1 is clearly able to convert crocetin dialdehyde into *trans*- and *cis*-crocin, while the remaining five ALDH enzymes have no obvious activity [8]. The third step is catalyzed by UDP-glucosyl transferases (UGTs), which glycosylate crocetin to produce the five types of crocin (Figure 1), which have different degrees of glycosylation [64,100]. In higher plants, glycosylation is a key process for converting insoluble secondary metabolites into soluble and stable storage forms [6]. In *G. jasminoides*, the carboxyl groups at each end of the crocetin carbon chain are first glycosylated by Gj-UGT75L6; this generates crocin V, which has a glycosyl group at one end, and crocin III, which has glycosyl groups at both ends [18]. The glycosyltransferase enzymes of Gj-UGT94E5 perform further glycosylation reactions on the 6-hydroxyl of the glucose moiety to produce the remaining three crocins [18]. However, in vitro experiments found that Gj-UGT75L6 has only weak glycosylation activity, while Gj-UGT94E5 has no catalytic activity [7]. The conversion efficiency into crocin of both enzymes in *E. coli* is low and results in different degrees of glycosylation. The Gj-UGT94E5 and Gj-UGT75L6 also lack the ability to produce crocin with a specific level of glycosylation [7,101]. At the same time, the glycosyltransferases of Bc-GTA from *Bacillus cereus* WQ9-2 and the glycosyltransferases of Bs-GT, Bs-YdhE, Bs-YjiC, and Bs-Yojk from *Bacillus subtilis* 168, i.e., a total of six microbially derived glycosyltransferases, can also produce low levels of crocin [7,101]. Recent examples of key enzymes involved in heterologous crocin biosynthesis are shown in Table 2. 

In December of this year, crocin produced the highest heterologous yield on record. Chai et al. [62] successfully introduced *Ps-CrtZ*, *Cs-CCD2*, and *Sca-LD* genes into *Saccharomyces cerevisiae*, resulting in a yield of 6.278 mg/L of crocetin via fermentation. Wang et al. [7] introduced *Cs-CCD2L* and different *UGTs* genes into *Escherichia coli* and synthesized crocetin of 4.42 mg/L. Xie et al. [102] for the first time transformed *Gj-ALDH2C3* and glycosyltransferase *Gj-UGT74F8* and *Gj-UGT94E13* genes into *Nicotiana benthamiana*, resulting in a yield of 105.8945 mg/g DW of crocins, of which crocin I and crocin II accounted for 99%. It is the highest recorded heterologous synthesis of crocin.

**Table 2:** Key enzymes involved in heterologous crocin biosynthesis.

Enzymes	Name	Source	Host	Production	Yield	Year	Ref.
CCDs	ZCD1	*C. sativus* L.	*C. vulgaris*	Crocetin	Detectable	2016	[103]
	Cs-CCD2L	*C. sativus* L.	*E. coli*	Crocetin	4.42 mg/L	2019	[7]
	Fh-CCD7	*F. hybrida*	*E. coli*	Crocetin	Detectable	2020	[104]
	Cs-CCD2L	*C. sativus* L.	*S. cerevisiae*	Crocetin	12.43 ± 0.62 mg/L	2020	[105]
	Cs-ZCD	*C. sativus* L.	*D. salina*	Crocetin	Detectable	2020	[104]
	Bd-CCD4.1	*B. davidii*	*N. glauca*	Crocins	2.18 ± 0.23 mg/g DW	2020	[52]
	Bo-CCD4.3	*B. orellana*	*S. lycopersicum*	Crocins	0.1 mg/g DW	2021	[106]
	Gj-CCD4a	*G. jasminoides*	*N. glauca*	Crocins	1.6 mg/g DW	2022	[98]
	Cs-CCD2L	*C. sativus* L.	*S. lycopersicum*	Crocins	14.48 ± 0.18 mg/g DW	2022	[107]
ALDHs	Syn-ALD	*Synechocystis* sp. PCC6803	*S. cerevisiae*	Crocetin	6.278 mg/L	2017	[62]
	pTrc-ALD8	*N. crassa*	*E. coli*	Crocetin	4.42 mg/L	2019	[7]
	Gj-ALDH2C3	*G. jasminoides*	*N. benthamiana*	Crocins	105.8945 mg/g DW	2023	[102]
UGTs	Cs-UGT2	*C. sativus* L.	*E. coli*	Crocetin	6.278 mg/L	2004	[62]
	Cs-UGT74AD1	*C. sativus* L.	*E. coli*	Crocetin	6.278 mg/L	2018	[62]
	Gj-UGT75L6	*G. jasminoides*	*E. coli*	Crocetin	4.42 mg/L	2019	[7]
	Gj-UGT94E5	*G. jasminoides*	*E. coli*	Crocetin	4.42 mg/L	2019	[7]
	Bs-YdhE	*B. subtilis*	*E. coli*	Crocetin	4.42 mg/L	2019	[7]
	Bs-YjiC	*B. subtilis*	*E. coli*	Crocetin	4.42 mg/L	2019	[7]
	Bs-Yojk	*B. subtilis*	*E. coli*	Crocetin	4.42 mg/L	2019	[7]
	Gj-UGT74F8	*G. jasminoides*	*E. coli*	Crocin III, crocin V	33.05 mg/L (66.1%)	2020	[61]
	Gj-UGT94E13	*G. jasminoides*	*E. coli*	Crocins	29.8 mg/L (59.6%)	2020	[61]
	Bs-GT	*B. subtilis* 168	*E. coli*	Crocins	476.8 mg/L (81%)	2018	[101]
	Cs-UGT74AD1	*C. sativus* L.	*S. lycopersicum*	Crocins	14.48 ± 0.18 mg/g DW	2022	[107]
	Cs UGT709G1	*C. sativus* L.	*S. lycopersicum*	Crocins	14.48 ± 0.18 mg/g DW	2022	[107]
	Gj-UGT74F8	*G. jasminoides*	*N. benthamiana*	Crocins	105.8945 mg/g DW	2023	[102]
	Gj-UGT94E13	*G. jasminoides*	*N. benthamiana*	Crocins	105.8945 mg/g DW	2023	[102]

## 5. Heterologous Production of Crocins in Different Species

**Figure 4:**
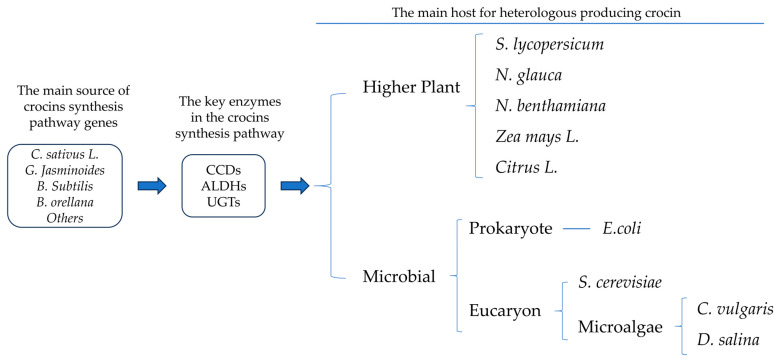
Basic logic of heterologous synthesis of crocin by genetic engineering and the common host.

As a high-value carotenoid, crocins have great potential in pharmacology. Nowadays, many key enzymes in the crocins synthesis pathway have been widely revealed by transcriptomic and dynamic metabolomics studies, while the traditional cultivation model cannot solve the crocins production problem in a short time. It may be a new direction to use genetic engineering technology to transform the key enzymes of the synthetic pathway of crocetin to produce crocetin in species with a high yield of β-carotene or the potential to synthesize β-carotene or crocetin (Figure 4). According to available reports, many microorganisms have been successfully transformed to synthesize crocetin or crocetin, including *E. coli* [7], *yeast* [62], *Nicotiana glauca* [108], *Chlorella vulgaris* [109], and *Dunaliella salina* [104]. Recently, the transient transformation of *N. benthamiana* to synthesize crocins was reported [102]. The above cases provide solid theoretical support and practical basis for further heterologous production of crocin. Below, these cases are divided into higher plant hosts and microbial hosts, which are divided into *E. coli*, yeast, and microalgal (Figure 4).

### 5.1. Biosynthesis of Crocins in Higher Plant Hosts

In plants, *β*-carotene can be converted into other carotenoids to meet particular needs, especially in plants that utilize carotenoids to reduce photooxidative damage [14,107]. Heterologous production of crocins in plants has the advantage of requiring the introduction of only one or two genes since the other genes from the carotenoid pathway are already present [98]. Higher plant cells have abundant *β*-carotene storage capacity, a complete carotenoid synthesis system, and a complete endomembrane system and thus are ideal host cells for heterologous production of crocin [107]. In addition to *C. sativus*, crocin is also found in a variety of plants outside the Iridaceae, such as the flowers of *B. davidii* and the fruit of *G. jasminoides* [5,12]. However, such sources cannot meet the existing demand [49]. Therefore, researchers are investigating how to produce crocin efficiently in other higher plants.

In 1986, scientists first expressed human growth hormone in tobacco cells and proposed the concept of using plant cells as a platform for recombinant protein production [88]. After more than 30 years of development, plant hosts have become extremely diversified and include whole plants, various plant tissues, suspension cells, and other systems; in addition, there are multiple expression methods within each system [110,111]. Whole-plant cultivation requires special land and climatic conditions and is not suitable for rapid production of specific metabolites [112,113]. However, similarly to *E. coli* and *S. cerevisiae*, an isolated single-celled plant callus can be suspended and dispersed in liquid medium for rapid propagation and expression of products [114]. Therefore, suspension culture of plant cells has more prospects than whole-plant cultivation for large-scale industrial applications [115] and the production of high-value-added natural active products [110,116].

*N. benthamiana* is a plant that does not contain crocin itself, but when it is engineered with the appropriate CCD enzyme, it can overexpress upstream or downstream carotenoid-metabolizing genes, leveraging the crocin synthesis pathway. Zheng et al. [98] used a white citrus callus as host cells with a co-expression system comprising three genes, i.e., *Tp-CrtB*, *Os-BCH*, and *Gj-CCD4a*, and successfully constructed a non-green starch-rich tissue/organ expression platform for effective production of crocin. When the platform was introduced into the leaves of *N. benthamiana*, up to 1.6 mg/g dry weight crocin was obtained. It was found that Gj-CCD4a had higher substrate specificity and catalytic efficiency in the leaves, demonstrating that a single enzyme, Gj-CCD4a, could drive the synthesis of crocin [98]. Xie et al. [102] combined the strategy of fusion with the 2A polypeptide connection and successfully constructed a multi-gene vector containing four genes to *N. benthamiana*, which transformed GjCCD4a and two downstream glycosyltransferase genes Gj-UGT74F8 and Gj-UGT94E13, to achieve higher substrate conversion efficiency that solved the problem of the low proportion of the main active components crocin I and crocin II, especially crocin I, as evidenced in previous research for synthesizing crocin in transgenic tobacco, and transformed ALDH introduced into tobacco for the first time.

A related species, *N. glauca*, contains carotenoid pigments in its petals. Huang et al. [108] expressed the Bd-CCD4.1 enzyme from *B. davidii* constitutively in its petals and leaves and obtained 321.6 ± 21.3 µg/g and 302.7 ± 25.6 µg/g DCW crocin, respectively. Interestingly, in their transgenic lines expressing CsCCD2L, the difference in the accumulation of crocin between leaves and petals may have been due to the relatively higher accumulation of zeaxanthin or the tissue specificity of CCD in leaves [108]. Martí et al. used tobacco etch virus to drive the expression of Cs-CCD2L and Bd-CCD4.1 in *N. benthamiana* and found that only Cs-CCD2L could produce 2.18 ± 0.23 mg of crocins and 8.24 ± 2.93 mg of picrocrocin per gram (DCW) over 13 days. The study also found that CCD can intercept the metabolic flux in leaves and reduce the synthesis of lutein, which sharply increases the expression levels of phytoene and drives the carotenoid metabolic pathway in the direction of crocin synthesis [52]. 

Frusciante et al. introduced CCD2 into zeaxanthin-rich maize endosperm by *Agrobacterium*-mediated transient expression and found that, unlike in *E. coli*, where only crocetin dialdehyde could be detected, zeaxanthin was not only converted to crocin dialdehyde but also further oxidized to crocetin. This is likely because maize endosperm possesses an endogenous aldehyde dehydrogenase to facilitate the oxidation step. 

Due to the presence of vacuoles, plant cells are large compared with those of *E. coli* and *S. cerevisiae*. Accordingly, when using comparable culture volumes, it is difficult to improve production with plant cell cultures by increasing the number of cells. As a result, relatively low yields of recombinant protein product (0.01 to 10 mg/L) are achieved in plant cell systems [110,111]. In addition, not all plant species can be adapted to suspension cell culture in a fermenter due to the presence of exogenous plant enzymes [117].

### 5.2. Microbial Biosynthesis of Crocins

Another approach to the production of crocins is combinatorial biosynthesis, which consists of combining enzyme-encoding genes from different species and designing a new set of gene clusters to produce bioactive compounds in a heterologous host. The commonly used microbial hosts for crocin production are *E. coli* among the prokaryotes and the yeast *S. cerevisiae* among the eukaryotes [7,62]. It is very important to select a suitable host organism for the optimization of product yield and quality, and there are pros and cons for both bacteria and yeast in this context. Bacterial hosts have a short life cycle, offer easy genetic manipulation and handling, and have a higher growth rate and excellent potential for protein and enzyme overexpression; however, they are not as beneficial for large proteins and proteins requiring post-translational modifications, which may be essential for correct folding and functional activity [115]. The *S. cerevisiae* is also well characterized and easy to grow and manipulate like *E. coli* but in addition can express proteins with appropriate post-translational modifications and offers better expression of membrane proteins; moreover, it has food-grade status (generally recognized as safe; GRAS) [118]. However, it results in lower yields than bacteria and can add a large number of mannose residues to recombinant proteins, resulting in protein misfolding and problems with activity [117]. 

All in all, heterologous production of crocins in microorganisms is highly advantageous since they can grow on inexpensive substrates and, compared to plants, are easier to manipulate and have very rapid production cycles, allowing crocins to be produced faster and possibly in larger amounts [119]. Thus, taking into consideration all the pros and cons, the commercial application of heterologous production of crocins by microorganisms is the more attractive route.

#### 5.2.1. Biosynthesis of Crocins in *E. coli*

Wild-type *E. coli* does not have the ability to synthesize carotenoids itself, but after metabolic engineering, it can successfully synthesize *β*-carotene and various other carotenoids [120,121,122]. Therefore, *E. coli* has the potential to synthesize crocin after appropriate pathway modification.

These were the first reports to demonstrate functional expression of a carotene gene cluster in *E. coli*: Perry et al. [123] and Tuveson et al. [124] introduced a 12.4 kb carotene gene cluster from *Erwinia herbicola* (reclassified as *Pantoea agglomerans*) into *E. coli* and successfully produced yellow pigmentation. Misawa et al. [125] isolated a 6.9 kb yellow-pigment-producing gene cluster fragment from the above genome segment and found six open reading frames: *CrtE, CrtI, CrtB, CrtX, CrtY*, and *CrtZ*. It was confirmed that the yellow substance produced using this gene cluster was zeaxanthin and that the recombinant *E. coli* could also synthesize phytoene, lycopene, *β*-carotene, zeaxanthin, and basic carotenoids with GGPP as a substrate. In recent years, *E. coli* has often been used as a host strain for the production of various carotenoids, which thus provides a theoretical basis for the heterologous synthesis of crocin in prokaryotes. 

In terms of crocins synthesis, Wang et al. [7] introduced the *Cs-CCD2L* gene of *C. sativus* and the glycosyltransferases Gj-UGT94E5 and Gj-UGT75L6 of *G. jasminoides* into *E. coli*, which was then capable of producing zeaxanthin and crocetin dialdehyde. This strain was able to produce crocetin after further engineering with the *pTrc-ALD8* gene from *Neurospora crassa*. Finally, the glycosyltransferases of Bs-YjiC, Bs-YdhE, and Bs-YojK were introduced into the expression system to obtain crocin V with a yield of 4.42 mg/L. This was the first time that a heterologous crocetin and crocin synthesis pathway was successfully constructed in *E. coli* [7]. Ding et al. [101] successfully mined two microbially derived glycosyltransferases with higher heterologous production and catalytic efficiency to improve crocin production. It was found that Bs-GT glycosyltransferase from *Bacillus subtilis* 168 could achieve a maximum crocetin glycosylation conversion efficiency of 81.9% and a yield of 476.8 mg/L crocin V and crocin III. Bc-GTA showed a much lower conversion efficiency and specificity than Bs-GT [101]. Pu et al. [61] found that *G.-jasminoides*-derived Gj-UGT74F8 and Gj-UGT94E13 gave whole-cell biotransformation rates as high as 66.1% and 59.6% for 50 mg/L crocin, respectively, which was higher than was achieved using UGTs from microorganisms. By precisely controlling the glycosylation time course, a high concentration of crocin with a specific degree of glycosylation can be obtained. Further optimization of gardenia UGTs may provide a valuable tool for the industrial production of crocin [126]. At the same time, Pu et al. also found that the glucose content in the culture environment is one of the key factors for obtaining crocin. When the endogenous UDPG supply in engineered strains is insufficient for the efficient production of crocin, appropriate supplementation with a certain concentration of glucose can improve the catalytic activity of heterologously expressed UGTs to maintain efficient and sustainable production [61]. 

The synthesis of crocin in *E. coli* has been well studied. However, like other prokaryotes, the *E. coli* does not have a complex internal membrane system, as do eukaryotes. Thus, heterologous production of various eukaryotic enzymes in *E. coli* may result in differences in folding and functional group modification, which may in turn lead to reduced catalytic efficiency or no enzyme activity. Since *C. sativus* is a eukaryote, the synthesis of crocin involves the transfer of metabolites between multiple subcellular compartments, e.g., from plastids to vacuoles, and the cooperation of various related enzymes [8]. Lack of these enzymes or use of structurally defective enzymes may affect crocin production or produce toxic byproducts. Zheng et al. [98] obtained crocetin dialdehyde in vitro by incubating *β*-apo-8′-carotene as a substrate with crude lysates of *E. coli* cells that expressed Gj-CCD4a, showing that Gj-CCD4a expressed in *E. coli* has enzyme activity. However, although *E. coli* itself has no endogenous ALDs, it has been reported that the properties of ALDs from microbial sources expressed in *E. coli* are better than those of endogenous *C. sativus* ALDs, while other plant-derived ALDs are expressed at very low levels in *E. coli* [7]. Further investigation and optimization of candidate ALDs is required.

#### 5.2.2. Biosynthesis of Crocins in *S. cerevisiae*

On account of its GRAS status, *S. cerevisiae* is often used in the field of food processing. Unlike the bacterial model of *E. coli*, *S. cerevisiae* is a eukaryotic microorganism and thus has a complete set of intracellular membranes, including nuclear membranes and various organelle membranes, which are similar to those in plant and mammalian cells and provide a complete transcription, translation, and modification environment for foreign genes [127]. The various compartments in the cell interior can also provide transport and storage space for gene expression products and metabolites. Since *S. cerevisiae* does not have an endogenous biochemical pathway for the synthesis of carotenoids, it is necessary to redesign the enzymes of crocin synthesis that initiate the MVA pathway to increase the levels of substrates to those required by the downstream pathway [105,128]. Shimada et al. redirected the ergosterol biosynthetic pathway in *S. cerevisiae* by introducing three genes required for lycopene synthesis, namely *CrtE, CrtB*, and *CrtI,* and they were thus able to synthesize lycopene with a yield of 1.1 mg/g dry cell weight (DCW). Ergosterol is a type of isoprene that shares a precursor with *β*-carotene and can provide abundant substrate for the production of crocin [129]. Lv et al. [130] designed a dual-metabolic pathway in *S. cerevisiae* that simultaneously uses acetyl-CoA in the cytoplasm and mitochondria. In terms of improving the utilization rate of precursors and expanding the production of isoprene, it was shown that this dual-metabolic pathway has advantages over those that only use the mitochondrial pathway or the cytoplasmic pathway in recombinant strains.

When *Eu-CrtZ* was introduced into *S. cerevisiae*, along with knock-out of the genes *Lpp1* and *Dpp1*, which are responsible for directing farnesyl pyrophosphate towards ergosterol synthesis, Mei et al. [131] initially found that zeaxanthin production was only increased by a small amount, but a high yield of 96.2 mg/L of zeaxanthin was achieved when three copies of the GAL1 high-strength promoter were used. Improvement of Zeaxanthin Production by Multiple-Copy Integration of Eu-crtZ [89]. Enhancing zeaxanthin production in *Y. lipolytica* was achieved by integrating the Eu-crtZ gene, in which the gene led to the highest titer and content for producing the target molecule, the expression cassette, into the 26S rDNA region. Xie et al. [89] achieved a 4.02-fold increase in the titer of zeaxanthin and a 721% increase in the content of zeaxanthin than the single copy and achieved a 21.98 ± 1.80 mg/L zeaxanthin titer. This high-yield engineered strain was named *SyBE*-Sc0123Z020. Chai et al. [62] selected three key enzymes, namely CrtZ, CCD, and ALD, from different species for expression in the *S. cerevisiae* strain *SyBE*-SC0014CY06, which was capable of producing *β*-carotene. The best combination of the three genes was *Ps-CrtZ* from *Pantoea stewartii*, *Cs-CCD2L* from *C. sativus*, and *Syn-ALD* from *Synechocystis sp.* PCC6803, which together produced 0.633 mg/L crocin. Tan et al. [66] designed, optimized, and synthesized a new Cs-ALD enzyme and introduced it into *S. cerevisiae SyBE*-Sc02070187-189, which was then capable of producing zeaxanthin, obtaining a yield of 62.79 µg/g DCW crocetin dialdehyde. Song et al. [105] knocked out *CIT2* and *MLS1*, two genes that consume acetyl-CoA in the cytoplasm, and increased the production of lycopene by 50%. They then constructed a fusion enzyme composed of *Ps-CrtZ* and CsCCD2, which increased the concentration of crocin by 44%, yielding 12.43 ± 0.62 mg/L crocin, which was twice as high as that produced by the initial strain *SyBE*-Sc0123C050 [62]. The above examples suggest that crocin production in *S. cerevisiae* is feasible, and this could provide a safe and efficient route of crocin production in eukaryotes. 

However, *S. cerevisiae* contains five characterized endogenous ALDH genes and a large number of other endogenous ALDH genes that have not been fully characterized and are difficult to remove. These endogenous ALDH genes will seriously interfere with the expression and function of exogenous ALDH genes, significantly reducing crocin productivity [63]. Amplifying the copy number of exogenous ALDH genes in *S. cerevisiae* can competitively inhibit the expression and function of the endogenous ALDH genes, improving the expression and specificity of the exogenous ALDH genes, thereby increasing the production of crocetin [63]. When Chai et al. [63] used the multicopy plasmid pRS426 to increase the copy number of *Cs-CCD2L* and *Syn-ALD*, the production of crocin was further increased to 1.219 mg/L, which was twice the yield obtained with a single-copy plasmid.

#### 5.2.3. Biosynthesis of Crocins in Microalgal Hosts

Microalgae are microscopic photosynthetic eukaryotes that live in aquatic environments [132]. As single-celled organisms and the ancestors of land plants originating about 100 million years ago, microalgae nevertheless have a carotenoid synthesis pathway similar to that of higher plants [85,133]. Thus, homologs of CCD1, CCD7, CCD8, and NCED are present in microalgae such that heterologous synthesis of crocin from *β*-carotene is possible [85,134]. Indeed, the complex carotenoid metabolism system in microalgae can synthesize a variety of carotenoids that are found in land plants, such as lutein, astaxanthin, fucoxanthin, and *β*-carotene [135]. Based on the background of Chlamydomonas *β*-carotene synthesis pathway, it can greatly reduce the building line of the crocin synthesis pathway module and workload.

Microalgae are characterized by a fast growth rate, relatively easy modification of endogenous metabolic pathways, and a complement of silent genes or genes expressed at low levels; this simplifies their metabolic engineering for use as a crocin bioreactor [136]. Carotenoids from microalgae have already been used for commercial purposes. For example, *C. vulgaris* can use lycopene as a precursor for the synthesis of *β*-carotene, zeaxanthin, astaxanthin, and other substances under different culture conditions [136]. *D. salina*, which can survive in extremely high-salt environments, produces *β*-carotene naturally. One benefit of the high-salinity culture environment is that it can effectively inhibit contamination by other microorganisms, thereby reducing culture costs [137].

In the 1960s, *C. vulgaris* became the first single-celled green alga to be exploited on a large scale because of its simple structure, fast growth, and low maintenance costs [138]. *C. vulgaris* has been used as a cell factory and can synthesize various nutrients through photosynthesis; it is also capable of synthesizing proteins, carbohydrates, carotenoids, and lipids. Its protein content can be as high as 68%, and it is widely used in human health foods and additives as well as for animal feed in aquaculture [139,140,141,142]. However, unbalanced cellular metabolic fluxes and competition between intermediate and precursor metabolites are challenges for the heterologous expression of crocin in microalgae. Lycopene ε-cyclase (LCYE) is a crucial enzyme that cyclizes lycopene to *α*-carotene and provides a large pool of substrate for the synthesis of lutein [143]. The enzyme of LCYE is encoded by the *CvLCYE* gene, whose nucleotide sequence is highly conserved in a variety of green algae [109]. Overexpression of the *CvLCYE* gene can greatly improve lutein production in *C. vulgaris* [109]. By blocking or silencing the expression of *CvLCYE* gene, more lycopene can flow to *β*-carotene synthesis, thereby providing more substrate for the synthesis of crocin. 

Based on this characteristic of *C. vulgaris*, Lou et al. [103] used *Agrobacterium*-mediated transient expression of the *CrtRB* gene from *Haematococcus pluvialis* and the *ZCD1* gene from the stigma of *C. sativus* in *C. vulgaris* and successfully detected the accumulation of crocin. This was the first report to demonstrate crocin production in microalgae. ZCD1 is a 13-amino-acid mutant of Cs-ZCD, which originally lacked the residues and domains necessary for zeaxanthin dioxygenase activity; this modification restores the activity [96,103], which is important for modifying the weak activity of CCD and increasing the production of crocin.

*D. salina* is a free-moving, single-celled green microalga with flagella but without a rigid cell wall [144]. The intracellular glycerol content of *D. salina* is more than 50% its weight, which allows it to regulate the osmotic pressure by changing the intracellular glycerol concentration [145]. Therefore, it can survive in salt solutions of 0.5% to 35%, i.e., up to nearly saturated solutions [145]. It is one of the most salt-tolerant eukaryotes known [146]. The optimal-growth salt concentration range for *D. salina* is 1.0–2.0 M NaCl [147]. Under high salt-stress conditions, i.e., 3.0–4.0 M NaCl, the synthesis of chlorophyll and cell growth are inhibited [148]. However, when operating at optimal salt concentrations, contamination by most non-halotolerant bacteria or protists is minimal, thus reducing production costs and helping to maintain an axenic environment [149]. Compared with higher plants, microalgae grow fast. Most higher plants depend on photosynthesis for their growth and reproduction [145,150]. On the other hand, *D. salina* has the highest known content of *β*-carotene in the plant kingdom [151,152]. It is rich in lutein, zeaxanthin, and *β*-carotene, the latter of which accounts for 14% of DCW [153]. *D. salina* is one of the most widely used algal species for the commercial production of *β*-carotene, and it also has strong potential for crocin synthesis [144,154,155,156]. Due to their versatility in adapting to a variety of growing conditions and climates (e.g., glacial to tropical and freshwater to highly saline) and different pH values, microalgae show distinct advantages over higher plants, reducing the need for sophisticated culture equipment and thereby reducing costs. Microalgae generally have higher carotenoid contents than higher plants. The major carotenoids in *D. salina* include 9- or 9′-*cis*-*β*-carotene and all-*trans*-*β*-carotene, which is preferentially absorbed compared to the 9-*cis*-*β*-isomer [151]. Nevertheless, the 9-*cis*-*β*-isomer has a higher antioxidant activity due to the higher reactivity of the *cis* bond compared to the *trans* bond [151]. Among all natural sources studied to date, *D. salina* possesses the highest content of 9-*cis*-*β*-carotene, reaching levels of up to 100 g/kg of DCW [151,152]. This would provide a large substrate pool for the production of crocin by *D. salina* [157]. The relative carotenoid content (% of total carotenoids) of octahydro-lycopene increased more than 48-fold in *D. salina* after treatment with mitogenic inhibitors (propyzamide and chlorpropham) for 10 h [157]. The production of lycopene and *β*-carotene was also significantly increased after exposure to red light. This is due to the accumulation of the more readily degraded 9-cis *β*-carotene under high-intensity red-light conditions; such conditions are associated with high rates of photooxidation, which in turn increases the activity of *β*-carotene isomerases, the gene transcripts of which are induced by light stress [158]. These characteristics of *D. salina* provide some conditions for the synthesis of crocin by transgenic technology.

By mining the transcriptome and genome of *D. salina* using deep sequencing, Lou et al. [159] found that, under high-light and high-salinity stress, *D. salina* activates an endogenous miRNA, *m0533-3p*, which in response to the stress signals inhibits malate dehydrogenase. This is likely to lead to a reduced flow of acetyl-CoA into the tricarboxylic acid cycle and instead greater participation of acetyl-CoA in the synthesis of GGPP, with a concomitant increase in *β*-carotene levels. However, as salt concentration increases, *D. salina* is more inclined to divert *β*-carotene to *α*-ionone and *β*-ionone synthesis to improve stress resistance, resulting in a decrease in *β*-carotene reserves, thus affecting the conversion efficiency of crocin [160,161,162]. Therefore, to balance these two opposing fluxes, the optimal salt concentration for *D. salina* should be 1.5 M NaCl [160,161]. Hou [104] introduced *CrtRB*, *Cs-ZCD*, and *CCD2* as target genes into *D. salina* by the glass-bead method and successfully detected trace amounts of crocetin dialdehyde.

*D. salina* has many applications in the pharmaceutical, nutraceutical, and cosmeceutical industries. However, although there are thorough and comprehensive research methods for using microalgae to produce other carotenoid products, they are still in the initial stages as hosts for the production of crocin; still, they have great potential for this application [42,163]. Nevertheless, it will not be enough to identify and modify the key enzymes in engineered pathways; there will also be a requirement for increased investment in the optimization of algal strains and for further investigation and optimization of culture conditions, methods of exogenous gene transformation, and the selection of transcription and translation-related factors [164].

## 6. Future Perspectives

Because of their potent biological activities with applications in the medical, food, and nutraceutical sectors, crocins are in great demand. Recently developed biological tools and techniques are helping to produce this bioactive plant product in microbial hosts at low cost and with short production times. Despite the lack of crocin biosynthetic genes in microbial hosts, they are an excellent alternative source for the large-scale production of crocins because of the availability of metabolic engineering and synthetic biology approaches. In the last decade, there have been remarkable advances in understanding the biosynthetic pathway of crocin production in *C. sativus* and its heterologous production in *E. coli.* However, the overall crocin production level is still not adequate to meet demand despite all the recent innovations. Therefore, further efforts aimed at exploiting new heterologous hosts and finding the best synthetic enzymes and plasmids are needed. Each gene involved in the biosynthetic pathway should be optimized to improve enzyme activity in the respective hosts to supply sufficient precursors and regulate the concentration of crocins inside the cell, all of which should help to increase production. In December of this year, crocin produced the highest heterologous yield on record, which included the highest yield of 4.42 mg/L of crocetin in *E. coli*, the highest yield of 6.278 mg/L of crocetin in *S. cerevisiae*, and the highest yield of 105.8945 mg/g DW of crocetin in *N. benthamiana*. Microbial production of crocins is still at an early stage and is limited by the identification of some crocin synthases. More intense investigations should allow the identification of novel enzymes that produce high yields of crocins that are found or not in nature and that may have significant commercial value. The huge accumulation of genome information from a wide variety of organisms and bioinformatic prediction of catalytic properties of gene products will allow the combination of the best enzymes to generate novel biosynthetic pathways for crocin production in various host organisms. Therefore, there should be a focus on a combinatorial approach where metabolites and precursors are directed towards crocin production. Moreover, a comprehensive understanding of the synthetic and molecular biology of each component involved in the biosynthesis of crocins—at the whole-genome, transcriptome, proteome, and metabolome levels—will help to increase yields. 

The crocin titers and yields obtained so far using microorganism are very promising, and we believe that, using synthetic biology approaches and metabolic engineering tools in *D. salina*, these can be further improved to make heterologous production competitive with the current process of extraction from plants. As mentioned above, heterologous production of crocins is preferable to extraction from plants, as the former can be easily controlled in a bioreactor and is not subject to unpredictable factors such as adverse weather, which can affect plant cultivation. Furthermore, in contrast to extraction from plants, heterologous production in microorganisms is not seasonal. In summary, we believe that after adequate optimization efforts, crocins can be produced by microorganisms in bioreactors, providing the same or larger yields as plant extraction in a shorter period of time and with a smaller footprint while using a process that is less expensive and more environmentally sustainable.

In conclusion, further research on crocin biosynthesis and metabolic engineering will contribute to the industrial production of crocins, which will not only bring huge economic benefits but also have beneficial effects on human health.

## Figures and Tables

**Table 1:** Levels of crocins in *C. sativus* in different countries.

Countries	Dry Weight	Ref.
Spain	0.85–32.40 mg/g	[65]
China	24.87 mg/g	[66]
Greece	26.60 mg/g	[66]
Morocco	29.00 mg/g	[67]
Iran	32.60 mg/g	[68]
Italy	49.80 mg/g	[68]
Nepal	89.00 mg/g	[69]

## Data Availability

Not applicable.

## References

[1] Schmidt M., Betti G., Hensel A. (2007). Saffron in phytotherapy: Pharmacology and clinical uses. Wien. Med. Wochenschr..

[2] Abdullaev F.I. (2002). Cancer chemopreventive and tumoricidal properties of saffron (*Crocus sativus* L.). Exp. Biol. Med..

[3] Melnyk J.P., Wang S., Marcone M.F. (2010). Chemical and biological properties of the world’s most expensive spice: Saffron. Food Res. Int..

[4] Pitsikas N. (2016). Constituents of Saffron (*Crocus sativus* L.) as Potential Candidates for the Treatment of Anxiety Disorders and Schizophrenia. Molecules.

[5] Guo Z., Li M., Li X., Wang P., Wang W., Du W., Yang Z., Chen S., Wu D., Tian X. (2022). Crocetin: A Systematic Review. Front. Pharmacol..

[6] Moraga A.R., Nohales P.F., Pérez J.A., Gómez-Gómez L. (2004). Glucosylation of the saffron apocarotenoid crocetin by a glucosyltransferase isolated from *Crocus sativus* stigmas. Planta.

[7] Wang W., He P., Zhao D., Ye L., Dai L., Zhang X., Sun Y., Zheng J., Bi C. (2019). Construction of *Escherichia coli* cell factories for crocin biosynthesis. Microb. Cell Factories.

[8] Demurtas O.C., Frusciante S., Ferrante P., Diretto G., Azad N.H., Pietrella M., Aprea G., Taddei A.R., Romano E., Mi J. (2018). Candidate Enzymes for Saffron Crocin Biosynthesis Are Localized in Multiple Cellular Compartments. Plant Physiol..

[9] Buiatti S., Guglielmotti M., Bertin F., Bertoli S., Passaghe P. (2023). Use of Friulan saffron in the production of craft beer. Eur. Food Res. Technol..

[10] Mok I.-K., Nguyen T.T.H., Kim D.H., Lee J.W., Lim S., Jung H.-y., Lim T., Pal K., Kim D. (2020). Enhancement of neuroprotection, antioxidant capacity, and water-solubility of crocins by transglucosylation using dextransucrase under high hydrostatic pressure. Enzym. Microb. Technol..

[11] Carmona M., Zalacain A., Sánchez A.M., Novella J.L., Alonso G.L. (2006). Crocetin Esters, Picrocrocin and Its Related Compounds Present in *Crocus sativus* Stigmas and *Gardenia jasminoides* Fruits. Tentative Identification of Seven New Compounds by LC-ESI-MS. J. Agric. Food Chem..

[12] Ahrazem O., Zhu C., Huang X., Rubio-Moraga A., Capell T., Christou P., Gómez-Gómez L. (2022). Metabolic Engineering of Crocin Biosynthesis in Nicotiana Species. Front. Plant Sci..

[13] Fan M., Li N., Li Y., Qian H., Rao Z., Wang L. (2023). Evaluation of the fate of key bioactive pigments and physical properties of steamed bread fortified with Gardenia fruit pomace. Int. J. Food Sci. Technol..

[14] Rodriguez-Concepcion M., Avalos J., Bonet M.L., Boronat A., Gomez-Gomez L., Hornero-Mendez D., Limon M.C., Meléndez-Martínez A.J., Olmedilla-Alonso B., Palou A. (2018). A global perspective on carotenoids: Metabolism, biotechnology, and benefits for nutrition and health. Prog. Lipid Res..

[15] Avila-Sosa R., Nevarez-Moorillon G.V., Ochoa-Velasco C.E., Navarro-Cruz A.R., Hernandez-Carranza P., Cid-Perez T.S. (2022). Detection of Saffron’s Main Bioactive Compounds and Their Relationship with Commercial Quality. Foods.

[16] Ashktorab H., Soleimani A., Singh G., Amin A., Tabtabaei S., Latella G., Stein U., Akhondzadeh S., Solanki N., Gondré-Lewis M.C. (2019). Saffron: The Golden Spice with Therapeutic Properties on Digestive Dis eases. Nutrients.

[17] Moallem S.A., Afshar M., Etemad L., Razavi B.M., Hosseinzadeh H. (2016). Evaluation of teratogenic effects of crocin and safranal, active ingre dients of saffron, in mice. Toxicol. Ind. Health.

[18] Liu T., Yu S., Xu Z., Tan J., Wang B., Liu Y.-G., Zhu Q. (2020). Prospects and progress on crocin biosynthetic pathway and metabolic engineering. Comput. Struct. Biotechnol. J..

[19] Spence C. (2023). Saffron: The colourful spice. Int. J. Gastron. Food Sci..

[20] Ji A., Jia J., Xu Z., Li Y., Bi W., Ren F., He C., Liu J., Hu K., Song J. (2017). Transcriptome-Guided Mining of Genes Involved in Crocin Biosynthesis. Front. Plant Sci..

[21] Kyriakoudi A., Tsimidou M.Z. (2015). A Food-Grade Approach to Isolate Crocetin from Saffron (*Crocus sativus* L.) Extracts. Food Anal. Methods.

[22] Dai Z., Wang Y., Zhou Z., Li S., Zhang X. (2018). Synthetic Biology for Production of Plant-derived Natural Products. Bull. Chin. Acad. Sci..

[23] Belyagoubi L., Loukidi B., Belyagoubi-Benhammou N., Gismondi A., Di Marco G., D’Agostino A., Canini A., Benmahieddine A., Rouigueb K., Ben Menni D. (2021). Valorization of Algerian Saffron: Stigmas and Flowers as Source of Bioactive Compounds. Waste Biomass Valorization.

[24] Khadfy Z., Atifi H., Mamouni R., Jadouali S.M., Chartier A., Nehmé R., Karra Y., Tahiri A. (2023). Nutraceutical and cosmetic applications of bioactive compounds of Saffron (*Crocus sativus* L.) stigmas and its by-products. South Afr. J. Bot..

[25] Alavizadeh S.H., Hosseinzadeh H. (2014). Bioactivity assessment and toxicity of crocin: A comprehensive review. Food Chem. Toxicol..

[26] Singh D. (2015). Neuropharmacological Aspects of *Crocus sativus* L.: A Review of Preclin ical Studies and Ongoing Clinical Research. CNS Neurol. Disord.-Drug Targets.

[27] Kokkinaki F., Ordoudi S.A. (2023). Insights into the FTIR Spectral Fingerprint of Saffron (*Crocus sativus* L.) Stigmas After Gentle Drying Treatments. Food Bioprocess Technol..

[28] Mzabri I., Addi M., Berrichi A. (2019). Traditional and Modern Uses of Saffron (*Crocus sativus*). Cosmetics.

[29] Gresta F., Lombardo G.M., Siracusa L., Ruberto G. (2008). Saffron, an alternative crop for sustainable agricultural systems. A review. Agron. Sustain. Dev..

[30] Cardone L., Castronuovo D., Perniola M., Cicco N., Candido V. (2020). Saffron (*Crocus sativus* L.), the king of spices: An overview. Sci. Hortic..

[31] Manuello Bertetto A., Garau M., Ricciu R., Satta G., Chiappori P., Concu A. (2018). Human Energy Involved in Manual and Mechanically Facilitate Harvesting of Saffron Flowers. Advances in Service and Industrial Robotics, Proceedings of the 26th International Conference on Robotics in Alpe-Adria-Danube Regio, Torino, Italy, 21–23 June 2017.

[32] García-Rodríguez M.V., Moratalla-López N., Carrión C., López-Córcoles H., Alonso G.L. (2020). The Combined Effects of Vegetative Stage Corms, Ultra Low Oxygen Cooling Storage and Incubation Time on *Crocus sativus* L.. Agronomy.

[33] Siracusa L., Gresta F., Avola G., Albertini E., Raggi L., Marconi G., Lombardo G.M., Ruberto G. (2012). Agronomic, chemical and genetic variability of saffron (*Crocus sativus* L.) of different origin by LC-UV–vis-DAD and AFLP analyses. Genet. Resour. Crop Evol..

[34] Parizad S., Dizadji A., Habibi M.K., Winter S., Kalantari S., Movi S., Lorenzo Tendero C., Alonso G.L., Moratalla-Lopez N. (2019). The effects of geographical origin and virus infection on the saffron (*Crocus sativus* L.) quality. Food Chem..

[35] Bayat M., Rahimi M., Ramezani M. (2016). Determining the most effective traits to improve saffron (*Crocus sativus* L.) yield. Physiol. Mol. Biol. Plants.

[36] Ortiz R., Mihovilovich E., Campos H., Ortiz O. (2020). Genetics and Cytogenetics of the Potato. The Potato Crop: Its Agricultural, Nutritional and Social Contribution to Humankind.

[37] Kashtwari M., Mansoor S., Wani A.A., Najar M.A., Deshmukh R.K., Baloch F.S., Abidi I., Zargar S.M. (2022). Random mutagenesis in vegetatively propagated crops: Opportunities, challenges and genome editing prospects. Mol. Biol. Rep..

[38] Denham T., Barton H., Castillo C., Crowther A., Dotte-Sarout E., Florin S.A., Pritchard J., Barron A., Zhang Y., Fuller D.Q. (2020). The domestication syndrome in vegetatively propagated field crops. Ann. Bot..

[39] Xing Y., Hernandez Nopsa J.F., Andersen K.F., Andrade-Piedra J.L., Beed F.D., Blomme G., Carvajal-Yepes M., Coyne D.L., Cuellar W.J., Forbes G.A. (2020). Global Cropland Connectivity: A Risk Factor for Invasion and Saturation by Emerging Pathogens and Pests. BioScience.

[40] Salehi A., Shariatifar N., Pirhadi M., Zeinali T. (2022). An overview on different detection methods of saffron (*Crocus sativus* L.) adulterants. J. Food Meas. Charact..

[41] Petrakis E.A., Cagliani L.R., Polissiou M.G., Consonni R. (2015). Evaluation of saffron (*Crocus sativus* L.) adulteration with plant adulterants by 1H NMR metabolite fingerprinting. Food Chem..

[42] Wang Y., Han T., Zhang X.-G., Zheng C.-J., Rahman K., Qin L.-P. (2009). LC Fingerprint and Hierarchical Cluster Analysis of *Crocus sativus* L. from Different Locations in China. Chroma.

[43] Liu B., Dong Y., Yao C., Jiang F. (2022). Survey on Resource Development of Saffron in China. Chin. J. Mod. Appl. Pharm..

[44] Pfister S., Meyer P., Steck A., Pfander H. (1996). Isolation and Structure Elucidation of Carotenoid−Glycosyl Esters in Gardenia Fruits (*Gardenia jasminoides* Ellis) and Saffron (*Crocus sativus* Linne). J. Agric. Food Chem..

[45] Xiao W., Li S., Wang S., Ho C.-T. (2017). Chemistry and bioactivity of *Gardenia jasminoides*. J. Food Drug Anal..

[46] Guan S., Pu Q., Liu Y., Wu H., Yu W., Pi Z., Liu S., Song F., Li J., Guo D.-A. (2021). Scale-Up Preparation of Crocins I and II from *Gardenia jasminoides* by a Two-Step Chromatographic Approach and Their Inhibitory Activity Against ATP Citrate Lyase. Molecules.

[47] Ichi T., Higashimura Y., Katayama T., Koda T., Shimizu T., Tada M. (1995). Analysis of Crocetin Derivatives from Gardenia *Gardenia jasminoides* Ellis Fruits. Nippon. Shokuhin Kagaku Kogaku Kaishi.

[48] Guijarro-Díez M., Castro-Puyana M., Crego A.L., Marina M.L. (2017). Detection of saffron adulteration with gardenia extracts through the determination of geniposide by liquid chromatography–mass spectrometry. J. Food Compos. Anal..

[49] Diretto G., López-Jiménez A.J., Ahrazem O., Frusciante S., Song J., Rubio-Moraga Á., Gómez-Gómez L. (2021). Identification and characterization of apocarotenoid modifiers and carotenogenic enzymes for biosynthesis of crocins in *Buddleja davidii* flowers. J. Exp. Bot..

[50] Shindo K., Sakemi Y., Shimode S., Takagi C., Uwagaki Y., Hattan J.-I., Akao M., Usui S., Kiyokawa A., Komaki M. (2022). Changes of Crocin and Other Crocetin Glycosides in Saffron through Cooking Models, and Discovery of Rare Crocetin Glycosides in the Yellow Flowers of *Freesia hybrida*. Front. Nutr..

[51] Lautenschläger M., Lechtenberg M., Sendker J., Hensel A. (2014). Effective isolation protocol for secondary metabolites from Saffron: Semi-preparative scale preparation of crocin-1 and trans-crocetin. Fitoterapia.

[52] Martí M., Diretto G., Aragonés V., Frusciante S., Ahrazem O., Gómez-Gómez L., Daròs J.-A. (2020). Efficient production of saffron crocins and picrocrocin in *Nicotiana benthamiana* using a virus-driven system. Metab. Eng..

[53] Sarma K.S., Sharada K., Maesato K., Hara T., Sonoda Y. (1991). Chemical and sensory analysis of saffron produced through tissue cultures of *Crocus sativus*. Plant Cell Tissue Organ Cult..

[54] Sankari M., Hemachandran H., Anantharaman A., Babu S., Madrid R.R., Fulzele D.P., Siva R. (2016). Identifying a Carotenoid Cleavage Dioxygenase 4a Gene and Its Efficient *Agrobacterium*-Mediated Genetic Transformation in *Bixa orellana* L.. Appl. Biochem. Biotechnol..

[55] Du J., Shao Z., Zhao H. (2011). Engineering microbial factories for synthesis of value-added products. J. Ind. Microbiol. Biotechnol..

[56] Ro D.K., Paradise E.M., Ouellet M., Fisher K.J., Newman K.L., Ndungu J.M., Ho K.A., Eachus R.A., Ham T.S., Kirby J. (2006). Production of the antimalarial drug precursor artemisinic acid in engineered yeast. Nature.

[57] Lau W., Sattely E.S. (2015). Six enzymes from mayapple that complete the biosynthetic pathway to the etoposide aglycone. Science.

[58] Liu X., Cheng J., Zhang G., Ding W., Duan L., Yang J., Kui L., Cheng X., Ruan J., Fan W. (2018). Engineering yeast for the production of breviscapine by genomic analysis and synthetic biology approaches. Nat. Commun..

[59] Yan X., Fan Y., Wei W., Wang P., Liu Q., Wei Y., Zhang L., Zhao G., Yue J., Zhou Z. (2014). Production of bioactive ginsenoside compound K in metabolically engineered yeast. Cell Res..

[60] Hou M., Wang R., Zhao S., Wang Z. (2021). Ginsenosides in Panax genus and their biosynthesis. Acta Pharm. Sin. B.

[61] Pu X., He C., Yang Y., Wang W., Hu K., Xu Z., Song J. (2020). In Vivo Production of Five Crocins in the Engineered *Escherichia coli*. ACS Synth. Biol..

[62] Chai F., Wang Y., Mei X., Yao M., Chen Y., Liu H., Xiao W., Yuan Y. (2017). Heterologous biosynthesis and manipulation of crocetin in *Saccharomyces cerevisiae*. Microb. Cell Factories.

[63] Tan H.X., Chen X.H., Liang N., Chen R.B., Chen J.F., Hu C.Y., Li Q., Li Q., Pei W.Z., Xiao W.H. (2019). Transcriptome analysis reveals novel enzymes for apo-carotenoid biosynthesis in saffron and allows construction of a pathway for crocetin synthesis in yeast. J. Exp. Bot..

[64] Shan M., Yao M., Liang N., Wang H., Wu N., Wang Y., Xiao W., Yuan Y.-J. (2023). One-Pot Efficient Bioconversion of Crocetin from Zeaxanthin via a Dual-Enzyme System. ACS Sustain. Chem. Eng..

[65] Alonso G.L., Salinas M.R., Garijo J., Sánchez-Fernández M.A. (2001). Composition of crocins and picrocrocin from spanish saffron (*Crocus sativus* L.). J. Food Qual..

[66] Koulakiotis N.S., Gikas E., Iatrou G., Lamari F.N., Tsarbopoulos A. (2015). Quantitation of Crocins and Picrocrocin in Saffron by HPLC: Application to Quality Control and Phytochemical Differentiation from Other Crocus Taxa. Planta Medica.

[67] Gonda S., Parizsa P., Surányi G., Gyémánt G., Vasas G. (2012). Quantification of main bioactive metabolites from saffron (*Crocus sativus*) stigmas by a micellar electrokinetic chromatographic (MEKC) method. J. Pharm. Biomed. Anal..

[68] Masi E., Taiti C., Heimler D., Vignolini P., Romani A., Mancuso S. (2016). PTR-TOF-MS and HPLC analysis in the characterization of saffron (*Crocus sativus* L.) from Italy and Iran. Food Chem..

[69] Li S., Shao Q., Lu Z., Duan C., Yi H., Su L. (2018). Rapid determination of crocins in saffron by near-infrared spectroscopy combined with chemometric techniques. Spectrochim. Acta Part A Mol. Biomol. Spectrosc..

[70] Havaux M. (2014). Carotenoid oxidation products as stress signals in plants. Plant J..

[71] Niu G., Guo Q., Wang J., Zhao S., He Y., Liu L. (2020). Structural basis for plant lutein biosynthesis from α-carotene. Proc. Natl. Acad. Sci. USA.

[72] Moreno J.C., Stange C., Wurtzel E.T. (2022). Chapter Nineteen-Heterologous complementation in bacteria for functional analysis of genes encoding carotenoid biosynthetic enzymes. Methods in Enzymology.

[73] Demurtas O.C., Nicolia A., Diretto G. (2023). Terpenoid Transport in Plants: How Far from the Final Picture?. Plants.

[74] Cichoński J., Chrzanowski G. (2022). Microalgae as a Source of Valuable Phenolic Compounds and Carotenoids. Molecules.

[75] Ma Y., Zu Y., Huang S., Stephanopoulos G. (2023). Engineering a universal and efficient platform for terpenoid synthesis in yeast. Proc. Natl. Acad. Sci. USA.

[76] Wang X., Dowd C.S. (2018). The Methylerythritol Phosphate Pathway: Promising Drug Targets in the Fight against Tuberculosis. ACS Infect. Dis..

[77] Pu X., Dong X., Li Q., Chen Z., Liu L. (2021). An update on the function and regulation of methylerythritol phosphate and mevalonate pathways and their evolutionary dynamics. J. Integr. Plant Biol..

[78] Tholl D., Schrader J., Bohlmann J. (2015). Biosynthesis and Biological Functions of Terpenoids in Plants. Biotechnology of Isoprenoids.

[79] Demurtas O.C., de Brito Francisco R., Diretto G., Ferrante P., Frusciante S., Pietrella M., Aprea G., Borghi L., Feeney M., Frigerio L. (2019). ABCC Transporters Mediate the Vacuolar Accumulation of Crocins in Saffron Stigmas. Plant Cell.

[80] Iorizzo M., Ellison S., Senalik D., Zeng P., Satapoomin P., Huang J., Bowman M., Iovene M., Sanseverino W., Cavagnaro P. (2016). A high-quality carrot genome assembly provides new insights into carotenoid accumulation and asterid genome evolution. Nat. Genet..

[81] Huang W., Lin Y., He M., Gong Y., Huang J. (2018). Induced High-Yield Production of Zeaxanthin, Lutein, and β-Carotene by a Mutant of *Chlorella zofingiensis*. J. Agric. Food Chem..

[82] Jiang Y., Chen Z., Tong Y., Wang P. (2021). Antidepressant mechanism and active compounds of saffron from network pharmacology study. Pak. J. Pharm. Sci..

[83] Tian Y., Rao S., Li Q., Xu M., Wang A., Zhang H., Chen J. (2021). The coloring mechanism of a novel golden variety in Populus deltoides based on the RGB color mode. For. Res..

[84] Liu M., Ding W., Yu L., Shi Y., Liu J. (2022). Functional characterization of carotenogenic genes provides implications into carotenoid biosynthesis and engineering in the marine alga *Nannochloropsis oceanica*. Algal Res..

[85] Gong M., Bassi A. (2016). Carotenoids from microalgae: A review of recent developments. Biotechnol. Adv..

[86] Zhang X., Wang X., Zhang Y., Wang F., Zhang C., Li X. (2023). Development of isopentenyl phosphate kinases and their application in terpenoid biosynthesis. Biotechnol. Adv..

[87] Caseiro M., Ascenso A., Costa A., Creagh-Flynn J., Johnson M., Simões S. (2020). Lycopene in human health. LWT.

[88] Tae Hyug J., Keunho J. (2013). Overexpression and Characterization of Lycopene Cyclase (CrtY) from Marine Bacterium *Paracoccus haeundaensis*. J. Microbiol. Biotechnol..

[89] Xie Y., Chen S., Xiong X. (2021). Metabolic Engineering of Non-carotenoid-Producing Yeast *Yarrowia lipolytica* for the Biosynthesis of Zeaxanthin. Front. Microbiol..

[90] Liang N., Yao M.-D., Wang Y., Liu J., Feng L., Wang Z.-M., Li X.-Y., Xiao W.-H., Yuan Y.-J. (2021). CsCCD2 Access Tunnel Design for a Broader Substrate Profile in Crocetin Production. J. Agric. Food Chem..

[91] Ahrazem O., Diretto G., Argandoña J., Rubio-Moraga Á., Julve J.M., Orzáez D., Granell A., Gómez-Gómez L. (2017). Evolutionarily distinct carotenoid cleavage dioxygenases are responsible for crocetin production in *Buddleja davidii*. J. Exp. Bot..

[92] Stelluti S., Grasso G., Nebauer S.G., Alonso G.L., Renau-Morata B., Caser M., Demasi S., Lumini E., Gómez-Gómez M.L., Molina R.V. (2024). Arbuscular mycorrhizal symbiosis modulates the apocarotenoid biosynthetic pathway in saffron. Sci. Hortic..

[93] Bouvier F., Suire C., Mutterer J.r.m., Camara B. (2003). Oxidative Remodeling of Chromoplast Carotenoids: Identification of the Carotenoid Dioxygenase CsCCD and CsZCD Genes Involved in Crocus Secondary Metabolite Biogenesis. Plant Cell.

[94] Wang Y., Li S., Zhou Z., Sun L., Sun J., Shen C., Gao R., Song J., Pu X. (2023). The Functional Characteristics and Soluble Expression of Saffron CsCCD2. Int. J. Mol. Sci..

[95] Castillo R., Fernández J.A., GómezGómez L. (2005). Implications of Carotenoid Biosynthetic Genes in Apocarotenoid Formation during the Stigma Development of *Crocus sativus* and Its Closer Relatives. Plant Physiol..

[96] Rubio A., Rambla J.L., Santaella M., Gómez M.D., Orzaez D., Granell A., Gómez-Gómez L. (2008). Cytosolic and plastoglobule-targeted carotenoid dioxygenases from *Crocus sativus* are both involved in beta-ionone release. J. Biol. Chem..

[97] Frusciante S., Diretto G., Bruno M., Ferrante P., Pietrella M., Prado-Cabrero A., Rubio-Moraga A., Beyer P., Gomez-Gomez L., Al-Babili S. (2014). Novel carotenoid cleavage dioxygenase catalyzes the first dedicated step in saffron crocin biosynthesis. Proc. Natl. Acad. Sci. USA.

[98] Zheng X., Mi J., Balakrishna A., Liew K.X., Ablazov A., Sougrat R., Al-Babili S. (2022). Gardenia carotenoid cleavage dioxygenase 4a is an efficient tool for biotechnological production of crocins in green and non-green plant tissues. Plant Biotechnol. J..

[99] Kirch H., Bartels D., Wei Y., Schnable P.S., Wood A.J. (2004). The ALDH gene superfamily of Arabidopsis. Trends Plant Sci..

[100] López-Jimenez A.J., Frusciante S., Niza E., Ahrazem O., Rubio-Moraga Á., Diretto G., Gómez-Gómez L. (2010). A New Glycosyltransferase Enzyme from Family 91, UGT91P3, Is Responsib le for the Final Glucosylation Step of Crocins in Saffron (*Crocus sativus* L.). Int. J. Mol. Sci..

[101] Ding F., Liu F., Shao W., Chu J., Wu B., He B. (2018). Efficient Synthesis of Crocins from Crocetin by a Microbial Glycosyltransferase from *Bacillus subtilis* 168. J. Agric. Food Chem..

[102] Xie L., Luo Z., Jia X., Mo C., Huang X., Suo Y., Cui S., Zang Y., Liao J., Ma X. (2023). Synthesis of Crocin I and Crocin II by Multigene Stacking in *Nicotiana benthamiana*. Int. J. Mol. Sci..

[103] Lou S., Wang L., He L., Wang Z., Wang G., Lin X. (2016). Production of crocetin in transgenic *Chlorella vulgaris* expressing genes crtRB and ZCD1. J. Appl. Phycol..

[104] Hou K. (2020). Construction and Identification of ZCD Transgenic *Dunaliella salina*. Master’s Thesis.

[105] Song T.Q., Wu N., Wang C., Wang Y., Chai F.H., Ding M.Z., Li X., Yao M.D., Xiao W.H., Yuan Y.J. (2020). Crocetin Overproduction in Engineered *Saccharomyces cerevisiaevia* Tuning Key Enzymes Coupled with Precursor Engineering. Front. Bioeng. Biotechnol..

[106] Frusciante S., Demurtas O.C., Sulli M., Mini P., Aprea G., Diretto G., Karcher D., Bock R., Giuliano G. (2021). Heterologous expression of *Bixa orellana* cleavage dioxygenase 4–3 drives crocin but not bixin biosynthesis. Plant Physiol..

[107] Ahrazem O., Diretto G., Rambla J.L., Rubio-Moraga Á., Lobato-Gómez M., Frusciante S., Argandoña J., Presa S., Granell A., Gómez-Gómez L. (2022). Engineering high levels of saffron apocarotenoids in tomato. Hortic. Res..

[108] Huang X., Morote L., Zhu C., Ahrazem O., Capell T., Christou P., Gómez-Gómez L. (2022). The Biosynthesis of Non-Endogenous Apocarotenoids in Transgenic *Nicotiana glauca*. Metabolites.

[109] Lou S., Lin X., Liu C., Anwar M., Li H., Hu Z. (2021). Molecular cloning and functional characterization of CvLCYE, a key enzyme in lutein synthesis pathway in *Chlorella vulgaris*. Algal Res..

[110] Schillberg S., Raven N., Spiegel H., Rasche S., Buntru M. (2019). Critical Analysis of the Commercial Potential of Plants for the Production of Recombinant Proteins. Front. Plant Sci..

[111] Karki U., Fang H., Guo W., Unnold-Cofre C., Xu J. (2021). Cellular engineering of plant cells for improved therapeutic protein production. Plant Cell Rep..

[112] Melis A. (2012). Photosynthesis-to-fuels: From sunlight to hydrogen, isoprene, and botryococcene production. Energy Environ. Sci..

[113] Lopes da Silva T., Moniz P., Silva C., Reis A. (2019). The Dark Side of Microalgae Biotechnology: A Heterotrophic Biorefinery Platform Directed to ω-3 Rich Lipid Production. Microorganisms.

[114] Georgiev M.I., Weber J., Maciuk A. (2009). Bioprocessing of plant cell cultures for mass production of targeted compounds. Appl. Microbiol. Biotechnol..

[115] Xu J., Zhang N. (2014). On the way to commercializing plant cell culture platform for biopharmaceuticals: Present status and prospect. Pharm. Bioprocess..

[116] Tiwari S., Verma P.C., Singh P.K., Tuli R. (2009). Plants as bioreactors for the production of vaccine antigens. Biotechnol. Adv..

[117] Jareonsin S., Pumas C. (2021). Advantages of Heterotrophic Microalgae as a Host for Phytochemicals Production. Front. Bioeng. Biotechnol..

[118] Ye M., Ye Y., Du Z., Chen G. (2021). Cell-surface engineering of yeasts for whole-cell biocatalysts. Bioprocess Biosyst. Eng..

[119] Rodrigues J.L., Prather K.L., Kluskens L.D., Rodrigues L.R. (2015). Heterologous production of curcuminoids. Microbiol. Mol. Biol. Rev..

[120] Misawa N., Misawa N. (2021). When Carotenoid Biosynthesis Genes Met *Escherichia coli*: The Early Days and These Days. Carotenoids: Biosynthetic and Biofunctional Approaches.

[121] Sandmann G., Misawa N. (2021). Carotenoid Production in *Escherichia coli*: Case of Acyclic Carotenoids. Adv. Exp. Med. Biol..

[122] Misawa N., Maoka T., Takemura M., Wurtzel E.T. (2022). Chapter Three—Carotenoids: Carotenoid and apocarotenoid analysis—Use of *E. coli* to produce carotenoid standards. Methods in Enzymology.

[123] Perry K.L., Simonitch T.A., Harrison-Lavoie K.J., Liu S.T. (1986). Cloning and regulation of *Erwinia herbicola* pigment genes. J. Bacteriol..

[124] Tuveson R.W., Larson R.A., Kagan J. (1988). Role of cloned carotenoid genes expressed in *Escherichia coli* in protecting against inactivation by near-UV light and specific phototoxic molecules. J. Bacteriol..

[125] Misawa N., Nakagawa M., Kobayashi K., Yamano S., Izawa Y., Nakamura K., Harashima K. (1990). Elucidation of the *Erwinia uredovora* carotenoid biosynthetic pathway by functional analysis of gene products expressed in *Escherichia coli*. J. Bacteriol..

[126] Chyau C.-C., Chiu C.-Y., Hsieh H.-L., Hsieh D.W.-C., Hsieh C.-R., Chang C.-H., Peng R.Y. (2022). High-Purity Preparation of Enzyme Transformed Trans-Crocetin Reclaimed from Gardenia Fruit Waste. Plants.

[127] Chu L.L., Huy N.Q., Tung N.H. (2023). Microorganisms for Ginsenosides Biosynthesis: Recent Progress, Challenges, and Perspectives. Molecules.

[128] Shimada H., Kondo K., Fraser Paul D., Miura Y., Saito T., Misawa N. (1998). Increased Carotenoid Production by the Food YeastCandida utilis through Metabolic Engineering of the Isoprenoid Pathway. Appl. Environ. Microbiol..

[129] Miura Y., Kondo K., Shimada H., Saito T., Nakamura K., Misawa N. (1998). Production of lycopene by the food yeast, *Candida utilis* that does not naturally synthesize carotenoid. Biotechnol. Bioeng..

[130] Lv X., Wang F., Zhou P., Ye L., Xie W., Xu H., Yu H. (2016). Dual regulation of cytoplasmic and mitochondrial acetyl-CoA utilization for improved isoprene production in *Saccharomyces cerevisiae*. Nat. Commun..

[131] Mei X., Chen Y., Wang R., Xiao W., Wang Y., Li X., Yuan Y. (2016). Engineered Yeast Cell for Producing Zeaxanthin. Chin. Biotechnol..

[132] Mata T.M., Martins A.A., Caetano N.S. (2010). Microalgae for biodiesel production and other applications: A review. Renew. Sustain. Energy Rev..

[133] Merchant S.S., Prochnik S.E., Vallon O., Harris E.H., Karpowicz S.J., Witman G.B., Terry A., Salamov A., Fritz-Laylin L.K., Maréchal-Drouard L. (2007). The Chlamydomonas genome reveals the evolution of key animal and plant functions. Science.

[134] Liang M.H., He Y.J., Liu D.M., Jiang J.G. (2021). Regulation of carotenoid degradation and production of apocarotenoids in natural and engineered organisms. Crit. Rev. Biotechnol..

[135] Novoveská L., Ross M.E., Stanley M.S., Pradelles R., Wasiolek V., Sassi J.-F. (2019). Microalgal Carotenoids: A Review of Production, Current Markets, Regulations, and Future Direction. Mar. Drugs.

[136] Lin J., Lee D., Chang J. (2015). Lutein production from biomass: Marigold flowers versus microalgae. Bioresour. Technol..

[137] Chuka-ogwude D., Nafisi M., Taher H., Ogbonna J.C., Moheimani N.R. (2022). Food waste digestate as a source of nitrogen for the cultivation of *Dunaliella salina*: Influence on growth and carotenogenesis under hyper osmotic stress. J. Appl. Phycol..

[138] Spolaore P., Joannis-Cassan C., Duran E., Isambert A. (2006). Commercial applications of microalgae. J. Biosci. Bioeng..

[139] Liu J., Chen F., Posten C., Feng Chen S. (2016). Biology and Industrial Applications of Chlorella: Advances and Prospects. Microalgae Biotechnology.

[140] Dantas D.M.M., Cahú T.B., Oliveira C.Y.B., Abadie-Guedes R., Roberto N.A., Santana W.M., Gálvez A.O., Guedes R.C.A., Bezerra R.S. (2021). *Chlorella vulgaris* functional alcoholic beverage: Effect on propagation of cortical spreading depression and functional properties. PLoS ONE.

[141] An B.K., Jeon J.Y., Kang C.W., Kim J.M., Hwang J.K. (2014). The Tissue Distribution of Lutein in Laying Hens Fed Lutein Fortified Chlorella and Production of Chicken Eggs Enriched with Lutein. Korean J. Food Sci. Anim. Resour..

[142] Saadaoui I., Rasheed R., Aguilar A., Cherif M., Al Jabri H., Sayadi S., Manning S.R. (2021). Microalgal-based feed: Promising alternative feedstocks for livestock and poultry production. J. Anim. Sci. Biotechnol..

[143] Ting L., Chunlei S., Zhibing G., Xianming S. (2009). Cloning and analysis of the gene encoding lycopene epsilon cyclase in Chlorella protothecoides CS-41. Acta Microbiol. Sin..

[144] Pourkarimi S., Hallajisani A., Alizadehdakhel A., Nouralishahi A., Golzary A. (2020). Factors affecting production of beta-carotene from *Dunaliella salina* microalgae. Biocatal. Agric. Biotechnol..

[145] Hosseini Tafreshi A., Shariati M. (2009). Dunaliella biotechnology: Methods and applications. J. Appl. Microbiol..

[146] Chen H.H., Liang M.H., Ye Z.W., Zhu Y.H., Jiang J.G. (2023). Engineering the β-Carotene Metabolic Pathway of Microalgae *Dunaliella* to Confirm Its Carotenoid Synthesis Pattern in Comparison to Bacteria and Plants. Microbiol. Spectr..

[147] García-González M., Moreno J., Manzano J.C., Florencio F.J., Guerrero M.G. (2005). Production of *Dunaliella salina* biomass rich in 9-cis-β-carotene and lutein in a closed tubular photobioreactor. J. Biotechnol..

[148] Liang M., Qv X., Chen H., Wang Q., Jiang J. (2017). Effects of Salt Concentrations and Nitrogen and Phosphorus Starvations on Neutral Lipid Contents in the Green Microalga *Dunaliella tertiolecta*. J. Agric. Food Chem..

[149] Borowitzka L., Borowitzka M. (1990). Commercial Production of β-Carotene by *Dunaliella Salina* in Open Ponds. Bull. Mar. Sci..

[150] Perez-Garcia O., Escalante F.M.E., de-Bashan L.E., Bashan Y. (2011). Heterotrophic cultures of microalgae: Metabolism and potential products. Water Res..

[151] Hsu Y., Tsai C., Chang W., Ho Y., Chen W., Lu F. (2008). Protective effects of *Dunaliella salina*—A carotenoids-rich alga, against carbon tetrachloride-induced hepatotoxicity in mice. Food Chem. Toxicol..

[152] Wolf L., Cummings T., Müller K., Reppke M., Volkmar M., Weuster-Botz D. (2021). Production of β-carotene with *Dunaliella salina* CCAP19/18 at physically simulated outdoor conditions. Eng. Life Sci..

[153] Sui Y., Muys M., Van de Waal D.B., D’Adamo S., Vermeir P., Fernandes T.V., Vlaeminck S.E. (2019). Enhancement of co-production of nutritional protein and carotenoids in *Dunaliella salina* using a two-phase cultivation assisted by nitrogen level and light intensity. Bioresour. Technol..

[154] Han S., Kim S., Lee C., Choi Y. (2019). Blue-Red LED wavelength shifting strategy for enhancing beta-carotene production from halotolerant microalga, *Dunaliella salina*. J. Microbiol..

[155] Ye Z., Jiang J. (2010). Analysis of an Essential Carotenogenic Enzyme: Zeta-Carotene Desaturase from Unicellular Alga *Dunaliella saline*. J. Agric. Food Chem..

[156] Xu Y.A., Ibrahim I.M., Wosu C.I., Ben-Amotz A., Harvey P.J. (2018). Potential of New Isolates of *Dunaliella salina* for Natural beta-Carotene Production. Biology.

[157] Xu Y., Harvey P.J. (2021). Mitosis Inhibitors Induce Massive Accumulation of Phytoene in the Microalga *Dunaliella salina*. Mar. Drugs.

[158] Xu Y., Harvey P.J. (2019). Red Light Control of β-Carotene Isomerisation to 9-cis β-Carotene and Carotenoid Accumulation in *Dunaliella salina*. Antioxidants.

[159] Lou S., Zhu X., Zeng Z., Wang H., Jia B., Li H., Hu Z. (2020). Identification of microRNAs response to high light and salinity that involved in beta-carotene accumulation in microalga *Dunaliella salina*. Algal Res..

[160] Liang M., Wu F., Liang Z., Chen H., Jiang J. (2020). Induction of carotenoid cleavage by salt stress and the effect of their products on cell growth and pigment accumulation in *Dunaliella* sp. FACHB-847. Algal Res..

[161] Elleuch F., Hlima H.B., Barkallah M., Baril P., Abdelkafi S., Pichon C., Fendri I. (2019). Carotenoids Overproduction in *Dunaliella* sp.: Transcriptional Changes and New Insights through Lycopene β Cyclase Regulation. Appl. Sci..

[162] Ramel F., Mialoundama A.S., Havaux M. (2012). Nonenzymic carotenoid oxidation and photooxidative stress signalling in plants. J. Exp. Bot..

[163] Torres-Tiji Y., Fields F.J., Mayfield S.P. (2020). Microalgae as a future food source. Biotechnol. Adv..

[164] Rammuni M.N., Ariyadasa T.U., Nimarshana P.H.V., Attalage R.A. (2019). Comparative assessment on the extraction of carotenoids from microalgal sources: Astaxanthin from *H. pluvialis* and β-carotene from *D. salina*. Food Chem..

